# Microfluidics sorting enables the isolation of an intact cellular pair complex of CD8^+^ T cells and antigen-presenting cells in a cognate antigen recognition-dependent manner

**DOI:** 10.1371/journal.pone.0252666

**Published:** 2021-06-14

**Authors:** Soichiro Kuwabara, Yoshihiko Tanimoto, Mie Okutani, Meng Jie, Yasunari Haseda, Yumi Kinugasa-Katayama, Taiki Aoshi

**Affiliations:** 1 Vaccine Dynamics Project, BIKEN Innovative Vaccine Research Alliance Laboratories, Research Institute for Microbial Diseases (RIMD), Osaka University, Suita, Osaka, Japan; 2 Department of Cellular Immunology, RIMD, Osaka University, Suita, Osaka, Japan; Universite Paris-Sud, FRANCE

## Abstract

Adaptive immune responses begin with cognate antigen presentation-dependent specific interaction between T cells and antigen-presenting cells. However, there have been limited reports on the isolation and analysis of these cellular complexes of T cell-antigen-presenting cell (T/APC). In this study, we successfully isolated intact antigen-specific cellular complexes of CD8^+^ T/APC by utilizing a microfluidics cell sorter. Using ovalbumin (OVA) model antigen and OT-I-derived OVA-specific CD8^+^ T cells, we analyzed the formation of antigen-specific and antigen-non-specific T/APC cellular complexes and revealed that the antigen-specific T/APC cellular complex was highly stable than the non-specific one, and that the intact antigen-specific T/APC complex can be retrieved as well as enriched using a microfluidics sorter, but not a conventional cell sorter. The single T/APC cellular complex obtained can be further analyzed for the sequences of T cell receptor Vα and Vβ genes as well as cognate antigen information simultaneously. These results suggested that this approach can be applied for other antigen and CD8^+^ T cells of mice and possibly those of humans. We believe that this microfluidics sorting method of the T/APC complex will provide useful information for future T cell immunology research.

## Introduction

T cells play important roles in various health conditions including infection, cancer, allergy, and autoimmunity [1–7]. Each T cell expresses a unique T cell receptor (TCR), which recognizes antigenic peptides presented on the major histocompatibility complex (MHC) of antigen-presenting cells (APCs). When T cell-mediated immune responses begin, T cells usually interact with APCs, especially dendritic cells, which are one of the most efficient APCs for naïve T cells. When T cells recognize the cognate antigen peptide on the MHC of the dendritic cell surface through the unique TCR, strong cellular interactions between the T cells and APCs can be observed *in vitro* [8] and *in vivo* [8–12]. After this antigen-specific T/APC interaction, the T cells are activated, and they differentiate into various effector T cells such as CTL, Th1, Th2, Th17, which have distinct functions depending on the context of the host immune conditions.

Although the importance of T cells in many diseases and host health is well recognized, the medical applications of directly targeting T cells as a major effector for disease prevention and therapy are relatively limited, as compared to approaches of targeting antibody responses in the host, such as vaccination [13]. However, recent advances in T cell immunology are generating chimeric antigen receptor-T and TCR-T adoptive transfer therapies for cancer [14, 15], which are T cell-targeted therapies. Another T cell-targeted approach is immune checkpoint blockade therapies for cancer, which have attained great clinical success recently [16].

In TCR-T therapy, precise information regarding the cognate antigen and cognate antigen-specific TCRα/TCRβ is necessary to avoid the side effects caused by cross-reactivity with the normal host tissues [17]. Many approaches have been attempted to overcome these issues, by mostly using multiplex tetramer assays including CyTOF [18] or DNA boarding [19] for sensitive detection. Multiplexed tetramer libraries have also been used to reveal the TCR fingerprint of a single T cell [20]. Additionally, multiple TCR sequences from single tetramer-stained T cells can also be determined [21]. In spite of using these sophisticated approaches, T cell-recognizing antigen determination is usually not easy [22], especially in case of an engineered TCR to a tumor. In addition, cross-reactive normal tissue antigen determination is sometimes very difficult and unpredictable before a clinical trial [23].

T/APC interaction has been studied using many methods, including microscopy for observation [24], flow cytometry for quantification [25, 26], and microfluidics chip for single-cell analysis [27, 28]. Yet, the T/APC interaction has not been utilized for detecting and analyzing T cell-recognizing antigen and TCRα/TCRβ information in the past. In this study, we aimed to isolate an intact T/APC complex and obtain the cognate antigen and TCRα/TCRβ information from such an isolated T/APC complex. We found that microfluidics-based cell sorting enabled the isolation of an antigen-specific T/APC complex, as well as, detailed analysis of this immunological cellular complex.

## Material and methods

### Mice

C57BL/6 mice (purchased from CLEA Japan, Inc.) and OT-I transgenic Rag1^-/-^ mice were kept in a humidity-, temperature-, and light cycle-controlled animal facility at the Research Institute for Microbial Diseases (RIMD), Osaka University. Six to ten week-old female mice were used for all the experiments. All the animal experiments were approved by the Animal Care and Use Committee of RIMD, and conducted according to the Regulations on Animal Experiments of Osaka University (permit number: Biken-AP-H26-12-2).

### Cell culture

BW5147 cells [AKR/J mouse (H-2k)-derived T cell lymphoma cell line; purchased from Japanese Collection of Research Bioresources Cell Bank, Japan; JCRB9002], and DC2.4 dendritic cell line [29] were cultured in R-10 complete medium [RPMI 1640 medium (Nacalai Tesque) supplemented with 10% heat-inactivated fetal bovine serum (FBS, Sigma-Aldrich) and 1% penicillin-streptomycin (Nacalai Tesque)]. The 293T cell line (human embryonic kidney-derived epithelial cells transformed with large T antigen, purchased from Riken, Japan; RCB2202) was cultured in D-10 complete medium [DMEM (Nacalai Tesque) supplemented with 10% FBS and 1% penicillin-streptomycin]. Each cell was grown in 10 cm polystyrene tissue culture dishes at 37°C in a 5% CO_2_ incubator. All the T cells were prepared from the spleen of OT-I and C57BL/6 mice. Splenocyte suspensions were prepared by pushing the spleens through a cell strainer (Corning). After treatment with ACK lysis buffer for 5 min at room temperature (RT) to remove the red blood cells, the splenocytes were washed twice and re-suspended in R-10 complete medium.

### Generation of H2K^b^- and OVA-H2K^b^-expressing BW5147 cells

BW5147 cells expressing H2K^b^ and OVA-H2K^b^ were generated using the retroviral vector system, as previously described [30, 31]. In brief, the full segment of H2K^b^ and OVA were amplified using PCR, and cloned into pMX retroviral vector at the BamHI and SalI restrictions sites. Retroviral supernatants were generated by transfecting the pCL-Eco retrovirus packaging vector and each pMX vector containing the gene of interest into the 293T cell line. After transduction with 8 μg/mL polybrene, the populations were cloned by limiting dilution or suspended in DMEM containing 1.5% methylcellulose. The highest expression of a single H2K^b^-BW5147 cell was selected with the help of an FITC anti-mouse H2K^b^ antibody (BioLegend). OVA-H2K^b^-BW5147 clones were co-cultured with splenocytes from an OVA-immunized mouse. The highest IFN-γ producing clone was selected after screening with a mouse IFN-γ enzyme-linked immunosorbent assay (mouse IFN-γ DuoSet^®^ ELISA, R&D Systems).

### Cell labeling and flow cytometry

OVA-H2K^b^-BW5147 cells, H2K^b^-BW5147 cells, OT-I splenocytes, and C57BL/6 splenocytes were stained for 30 min at 37°C using 20 μM of CMTMR (CellTracker^™^ Orange CMTMR Dye, Thermo Fisher Scientific), 50 μM of CMAC (CellTracker^™^ Blue CMAC Dye, Thermo Fisher Scientific), 1 μM of CFSE (CellTrace^™^ CFSE Cell Proliferation Kit, Thermo Fisher Scientific), and 2 μM of Far Red (CellTrace^™^ Far Red Cell Proliferation Kit, Thermo Fisher Scientific), respectively. Flow cytometry analysis was performed with NovoCyte^®^ (ACEA Biosciences), followed by sorting of the cells using FACS with an FACSAria^™^ II Cell Sorter (BD Biosciences) or On-chip Sort [32] (On-chip Biotechnologies) system. Cell sorting was performed using the following settings: FACSAria^™^ II, pressure = 70 psi; sorting speed = ca. 1000 events per second; nozzle diameter = 70 μm. On-chip Sort, pressure = 0.8 kPa (ca. 0.3 psi); sorting speed = ca. 100 events per second; fluidics channel diameter = 80 μm. Data analysis was performed using NovoExpress (Agilent) and FlowJo softwares (BD Biosciences).

### Cell-cell interaction analysis of the T/APC complex

Each cell type, stained with four different dyes, was mixed (OVA-H2K^b^-BW5147: 1×10^6^ cells, H2K^b^-BW5147: 1×10^6^ cells, C57BL/6 splenocytes: 1×10^6^ cells, OT-I splenocytes: 1×10^6^−1×10^1^ cells) and transferred into one well of a 96-well V-plate at RT and kept in the dark for 15 min. The plate was centrifuged at 400 × *g* for 5 min to reduce the total volume and promote further cell-cell interaction. The condensed cells in 70 μL of volume were then pipetted gently and analyzed for cellular complex formation using a flow cytometer, single-cell picking system, and fluorescence microscope.

### Antigen and TCR gene analysis of a single T/APC complex

OT-I splenocyte-CFSE-, C57BL/6 splenocyte-Far Red-, OVA-H2K^b^-BW5147-CMTMR-, and BW5147-H2K^b^-CMAC-stained cells were mixed in a ratio of 1:1:1:1 (each cell number = 1×10^5^ cells/50 μL) in a CO_2_ independent medium (Gibco) for 15 min at RT, and then centrifuged (1500 rpm, 5 min, RT). For single T/APC complex picking, the ASONE Cell Picking System (ASONE) [33, 34] was used, according to the manufacturer’s instructions (https://www.youtube.com/watch?v=zTC3pXJndeQ&feature=emb_logo). In brief, the cell pellet was resuspended five times by gently pipetting, then inoculated onto a microchamber slide (30 μm pore, 8.4×10^4^ cells/well, ASONE), and washed three to five times with PBS. The microchamber slide was pretreated with 1% (w/v) polyvinylpyrrolidone for 1 h at RT, and washed two times with distilled water. After that, the slide was scanned and the T/APC complex was checked visually using fluorescence; a single T/APC complex captured in the microchamber was picked up using the ASONE Cell Picking System [33, 34]. Each T/APC complex was transferred into a lysis buffer, followed by amplification of cDNA from the complex using the CellAmp^™^ Whole Transcriptome Amplification Kit (Real-Time) Ver.2 (Takara Bio), according to the manufacturer’s instructions. For a specific gene detection (OVA and GAPDH), real-time PCR was performed using the KAPA SYBR FAST qPCR Master Mix Kit (Kapa Biosystems) with LightCycler^®^ 480 (Roche), followed by separation of the amplicons using electrophoresis with a 2% agarose gel. The set of primers used have been described in Table 1. For the TCRVα and Vβ sequence, a multiplex nested PCR was performed using the Multiplex PCR Assay Kit Ver.2 (Takara Bio), with previously reported primers [35]. Next, the products were gel purified and cloned into a pMOD-T plasmid (Mighty TA-cloning Kit, Takara Bio). The sequences of the products were read using the M13 primer RV. The TCR CDR3 sequences obtained were analyzed with the IMGT online database (http://www.imgt.org/).

**Table 1:** Primers used in this study.

Primer name	Sequence	Comment
H2Kb_Fw	CGGGATCCACCATGGTACCGTGCACGCT	Creation of H2Kb-BW5147
H2Kb_Rv	CTGTCGACTCACGCTAGAGAATGAG	
OVA(pMX)_Fw	CGGGATCCACCATGGGCTCCATCGGTGC	Creation of OVA-H2Kb- BW5147
OVA(pMX)_Rv	CTGTCGACTTAAGGGGAAACACATC	
OVA_Fw	GTGGGACAATGAGCATGTTG	Detection of OVA antigen
OVA_Rv	TCCATCTTCATGCGAGGTAA	
GAPDH_Fw	GGTGAAGGTCGGTGTGAAC	Reference gene
GAPDH_Rv	GACTGTGCCGTTGAATTTG	

## Results

### Cellular interaction analysis between CD8^+^ T cells and antigen-presenting cells

**Fig 1:**
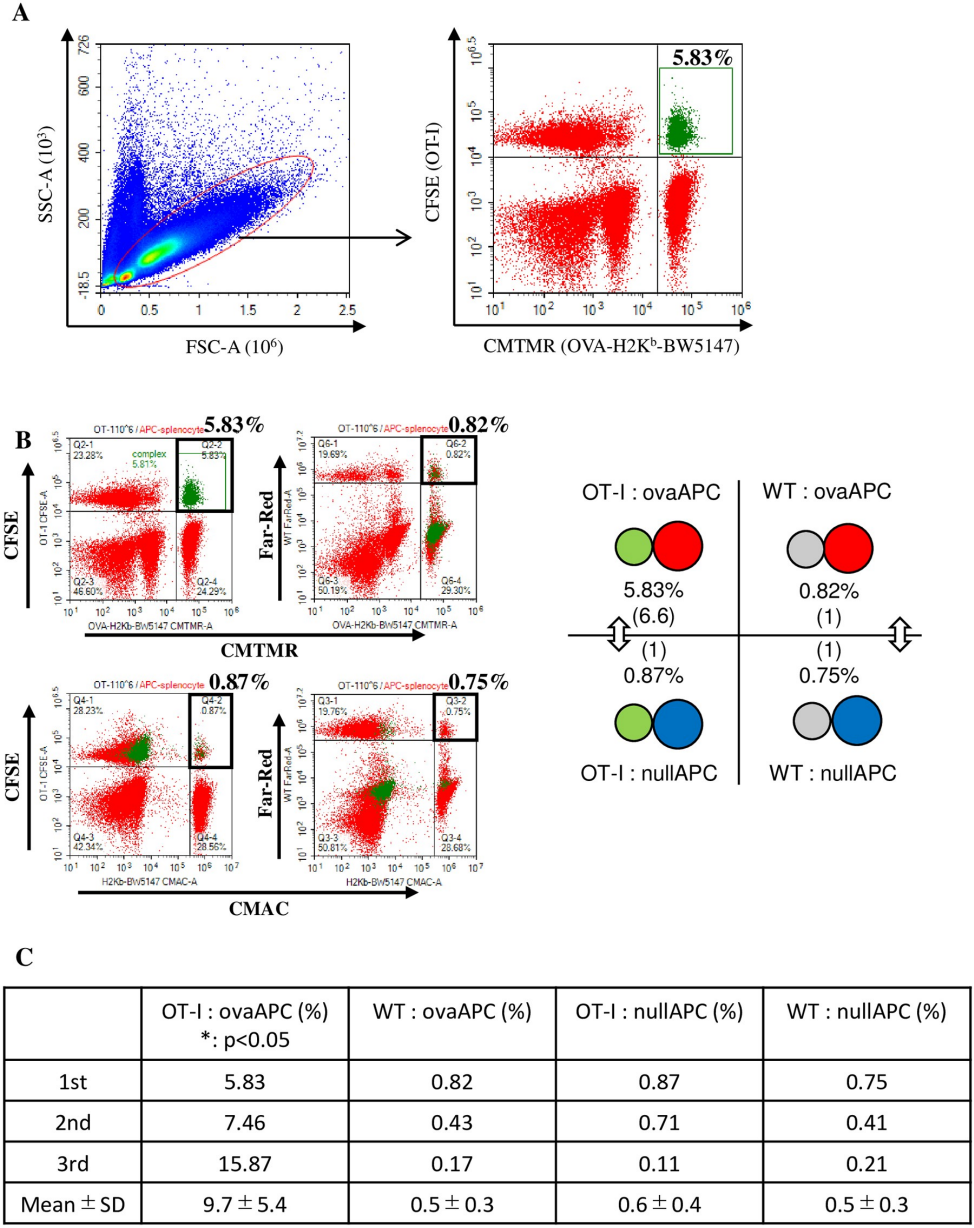
High frequency of cellular complex formation is dependent on the specific interaction between T and APC cells. **(A)** The cells were gated using FSC and SSC, following which the cellular complex formation was analyzed using a two-dimensional dot plot. CFSE and CMTMR double-positive fractions were derived from the cellular complex between OT-I and ovaAPC. This is a representative plot of three independent experiments. **(B)** Other combinations of each cellular complex were similarly analyzed using a conventional flow cytometer. Each bold black square shows the cellular complex of OT-I/ovaAPC (upper left), OT-I/nullAPC (lower left), WT/ovaAPC (upper right), and WT/nullAPC (lower right). Representative data of three independent experiments has been shown. In the right, four types of cellular complexes have been illustrated. **(C)** Percentages of each cellular complex in three similarly performed independent experiments [including data shown in (A) and (B)]. The row numbers indicate mean±SD of the cellular complex percentages. OT-I:ovaAPC complexes showed significantly higher complex formation than the other cellular complexes, when compared using standard t-test (p<0.05).

To study antigen-specific T/APC cellular interaction and complex formation at the cellular level, we used splenocytes from OT-I mice in Rag1 knockout background and C57BL/6J WT mice as the antigen-specific and non-specific T cells, respectively. CD8^+^ T cells from OT-I transgenic mice recognized OVA-derived peptide SIINFEKL (OVA_257-264_) bound to H-2K^b^ of the MHC class I molecule [36]. As an antigen non-expressing APC, we used the previously established H-2K^b^-expressing BW5147 cell line (H2K^b^-BW5147) [30, 31]. As OVA antigen-expressing APC, we used both OVA- and H-2K^b^-expressing BW5147 cells, which were generated through retroviral transduction of the OVA gene into the H2K^b^-BW5147 cell (OVA-H2K^b^-BW5147) (see Methods). To differentiate these cells, OVA-H2K^b^-BW5147 (ovaAPC), H2K^b^-BW5147 (nullAPC), OT-I, and C57BL/6 (WT) splenocytes were stained with the fluorescent dyes CMTMR, CMAC, CFSE, and Far Red, respectively. Stained cells of each cell type (1×10^6^) were mixed, followed by analysis of the percentage of CFSE/CMTMR (OT-I/ovaAPC) complexes (which indicated antigen-specific T/APC interaction) using conventional flow cytometry. One of the representative experimental results demonstrated that OT-I/ovaAPC complexes were detected in 5.83% of the total analyzed cells (Fig 1A; right). The other cellular complexes namely CFSE/CMAC (OT-I/nullAPC), Far Red/CMTMR (WT/ovaAPC), and Far Red/CMAC (WT/nullAPC), which indicated non-specific T/APC interactions, were detected in less than 1% of the total analysis (Fig 1B). This data was reproducible by three times, suggested that T/APC complex formation is significantly more frequent in antigen-specific cellular interactions, such as OT-I/ovaAPC, than in antigen-non-specific cellular interactions, namely OT-I/nullAPC, WT/ovaAPC, and WT/nullAPC (Fig 1C).

**Fig 2:**
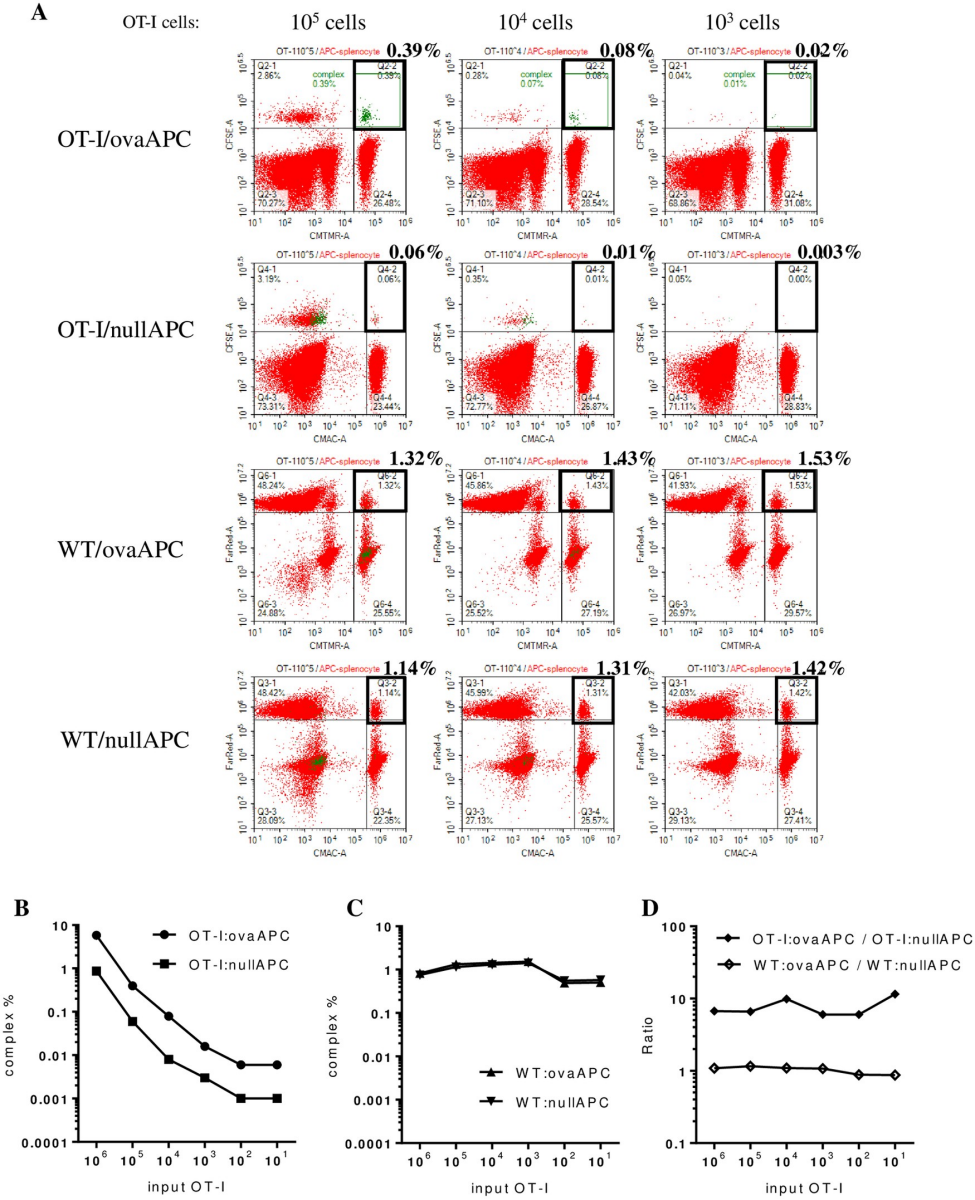
OT-I/ovaAPC complexes were more frequently observed than OT-I/nullAPC complexes, in a broad range of available OT-I cell numbers. **(A)** OT-I, ovaAPC, WT, and nullAPC were mixed as described in Fig 1, but only the OT-I cell numbers were serially decreased from 10^6^–10^1^, while the other cell number were kept constant at 10^6^, followed by analysis of each cellular complex formation as described in Fig 1. This figure shows only the panels for 10^5^, 10^4^, and 10^3^ cells. **(B)** Line graphs for the frequencies of OT-I/ovaAPC and OT-I/nullAPC complexes were plotted for input OT-I numbers from 10^6^ to 10^1^. In all the examined conditions, OT-I/ovaAPC complexes were 6 to 10 times more frequent than OT-I/nullAPC complexes. **(C)** Line graphs for the frequencies of WT/ovaAPC and WT/nullAPC complexes were plotted for input OT-I numbers from 10^6^ to 10^1^. **(D)** Line graph of the ratio between OT-I/ovaAPC and OT-I/nullAPC. All the data in this figure is representative of two independent experiment with similar results.

When the number of OT-I splenocytes was serially reduced to 1×10^5^, 1×10^4^, and 1×10^3^ cells, the percentage of antigen-specific OT-I/ovaAPC complexes decreased gradually to 0.39%, 0.08%, and 0.02%, respectively (Fig 2A; OT-I/ovaAPC), indicating that OT-I/ovaAPC complex formation is proportionally dependent on the available number of OT-I cells. Similarly, the OT-I/nullAPC complex formation also decreased gradually to 0.06%, 0.01%, and 0.003% (Fig 2A; OT-I/nullAPC), respectively. In contrast, other non-specific cellular complex formation between WT cells and ova/null APC was constant, at approximately 1.0%, and not influenced by the OT-I input cell numbers (Fig 2A; WT/ovaAPC and WT/nullAPC).

As expected, these quantitative analyses of up to 1×10^1^ OT-I cells revealed that the percentages of both OT-I/ovaAPC and OT-I/nullAPC decreased upon reducing the number of input OT-I cells; however, in all the conditions, the OT-I/ovaAPC to OT-I/nullAPC ratio was virtually constant (Fig 2B), between 6 and 10, irrespective of the input OT-I cell numbers (Fig 2D). In contrast, the percentage of WT/ovaAPC and WT/nullAPC did not change (Fig 2C), and the ratio was always approximately 1 (Fig 2D), suggesting that in these experimental conditions, approximately 1% of the cellular complex was formed in the absence of specific antigen recognition between the TCR and peptide/MHC. Taken together, these data indicated that the cognate antigen-dependent cellular interactions in OT-I/ovaAPC were approximately 6 to 10 times more frequent than the non-cognate cellular interactions, such as WT/ovaAPC and WT/nullAPC.

### Intact recovery of antigen-specific T/APC complex is achievable through microfluidics-based cell sorting

**Fig 3:**
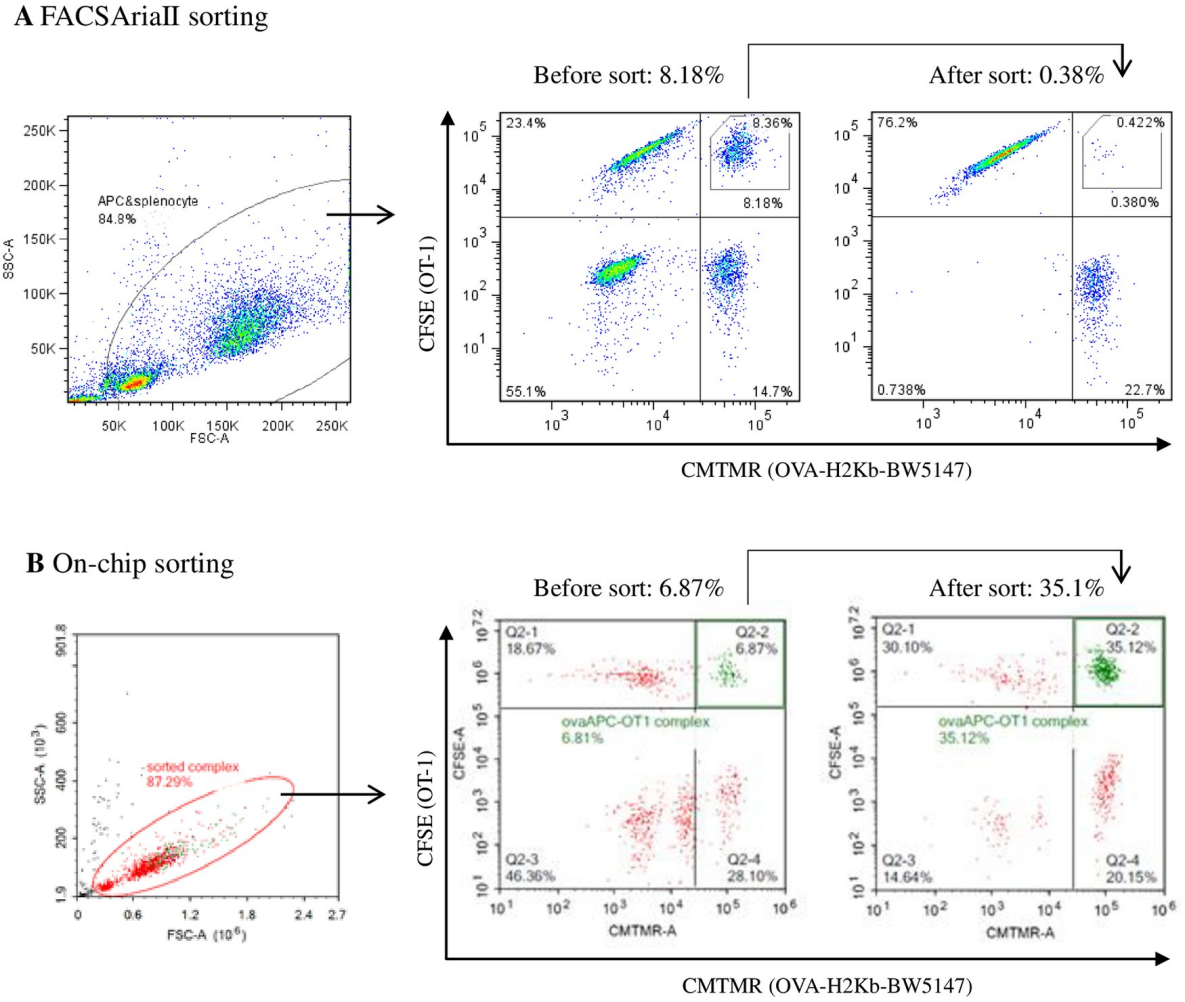
Specific cellular complex sorting using conventional sorter and microfluidics sorter. **(A)** OT-I/ovaAPC complexes (CFSE/CMTMR double-positive fraction) were sorted using the FACSAria^™^ II Cell Sorter. The sorted OT-I/ovaAPC complexes were also re-analyzed using FACSAria^™^ II Cell Sorter. **(B)** The samples were similarly sorted using the microfluidics sorter, On-chip Sort, and re-analyzed using the conventional flow cytometer, NovoCyte^®^.

Based on the above mentioned observation, we next examined whether the T/APC complex was recoverable using a regular cell sorter. Cellular complexes were similarly prepared by mixing the four different cell populations, as described above, followed by sorting of the OT-I/ovaAPC complex using the FACSAria^™^ II Cell Sorter with CFSE/CMTMR gating. The FACSAria^™^ II Cell Sorter was able to sort the OT-I/ovaAPC complex at 70,000 events/sec. However, re-analysis of the sorted cell population revealed that more than 95% of the OT-I/ovaAPC complex was disrupted into single cells during this sorting process (Fig 3A). This result suggests that the popularly used conventional cell sorter is not capable of isolating the intact antigen-specific T/APC cellular complex, which is desirable to obtain paired information about the specific TCRα/β and cognate antigen genes.

Based on this result, we also performed the same experiment with the recently developed microfluidics-based flow cytometer, On-chip Sort [32], which can sort cells without strong physical force such as shear stress. Upon using On-chip Sort, we successfully isolated the OT-I/ovaAPC complex, without losing out on the cellular interaction, resulting in further enrichment of the antigen-specific T/APC complexed cell population, owing to the above mentioned more stable cellular interaction of these antigen-specific cells (Fig 3B).

### Antigen-non-specific T/APC complex was not stably recoverable even with the microfluidics-based cell sorter

We also examined whether the antigen non-specific cellular complex was recoverable with the conventional and microfluidics cell sorters. With an experimental setting similar to that of Fig 3, the WT/ovaAPC complex was sorted using the FACSAria^™^ II Cell Sorter. Only 0.4% of the WT/ovaAPC complex was observed upon re-analysis post sorting (S1 Fig). This observed percentage was similar to the antigen-specific OT-I/ovaAPC complex recovery (0.4%) with the FACSAria^™^ II Cell Sorter (Fig 3A), suggesting that the T/APC complex was not recoverable at all using the conventional cell sorter, irrespective of antigen specificity.

**Fig 4:**
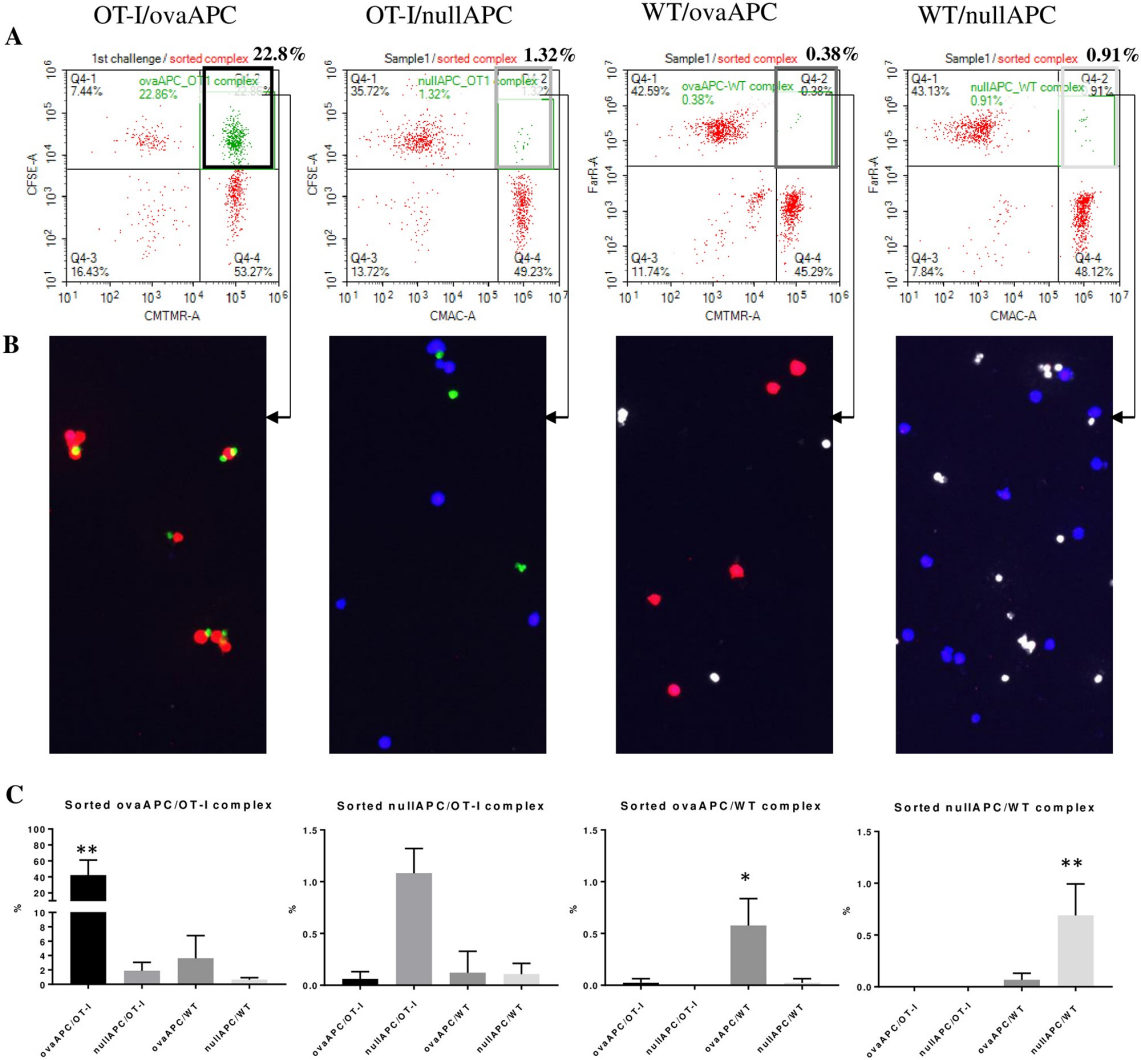
Non-specific cellular complex sorting using a microfluidics sorter. **(A)** The specific (OT-I/ovaAPC) and non-specific (OT-I/nullAPC, WT/ovaAPC, and WT/nullAPC) cellular complexes were sorted, following which the sorted complexes were re-analyzed for the enrichment of the sorted population using On-chip Sort. A representative plot of three independent experiments with similar results. Upper right area of each plot shows the percentage of intact T/APC complexes of the sorting target population. Representative data of at least three independent experiments with similar results has been shown. **(B)** Fluorescent micrographs of the sorted cellular complexes. **(C)** Each sorted complex contained a small amount of non-targeted populations. The sorted complexes were re-analyzed to check the concomitant existence of non-targeted complexes. Sorted OT-I/ovaAPC also contained a small amount of the other cellular complexes including OT-I/nullAPC, WT/ovaAPC, and WT/nullAPC. Similarly, the OT-I/nullAPC, WT/ovaAPC, and WT/nullAPC sorted populations also contained the other cellular complexes. The bar graph indicates mean±SD of three independent experiments. **p<0.005, *p<0.05 using ANOVA (with Kruskal-Wallis test).

Each of the four T/APC complexes, namely OT-I/ovaAPC, OT-I/nullAPC, WT/ovaAPC, and WT/nullAPC, were similarly sorted and re-analyzed using On-chip Sort. Similar to our previous result, the OT-I/ovaAPC complex was recovered with a high percentage (approximately 20–40%), while maintaining intact cellular complex formations (Fig 4A; OT-I/ovaAPC). On the other hand, the other complex populations of targets sorted were found to be intact in only around 1% of the re-analyzed total cells (Fig 4A; OT-I/nullAPC, WT/ovaAPC, WT/nullAPC). Of note, each sorted complex contained a small amount of irrelevant T/APC complexes; for example, the OT-I/ovaAPC sorted complex contained small amounts of OT-I/nullAPC, WT/ovaAPC, and WT/nullAPC (Fig 4C; OT-I/nullAPC). These irrelevant cellular complexes were mostly derived from the non-specific interaction observed in the Fig 1 experiment, although the targeted populations were substantially enriched upon using On-chip Sort (Fig 4C). OT-I/ovaAPC, WT/ovaAPC, and WT/nullAPC were significantly enriched, as compared to the other concomitant cellular populations (Fig 4C). Fluorescence micrograph of the sorted cells showed that the cellular complex formation of OT-I/ovaAPC was maintained, while the same was not maintain for the others, namely OT-I/nullAPC, WT/ovaAPC, and WT/nullAPC (Fig 4B). The sorted cellular complexes showed heterogeneity, especially OT-I/ovaAPC complexes (such as one ovaAPC with one OT-I cell, or one ovaAPC with two OT-I cells), which is probably due to the condensation of the cells post sorting enrichment (Fig 4B), which is required for re-analysis using FACS, to reduce the volume.

We performed a similar experiment using DC2.4 cells (C57/BL6 mouse-derived dendritic cell line) [29] instead of BW5147 cells as the APCs, to see the effect of costimulatory molecule (CD80 and CD86)-expressing APCs, because BW5147 cells do not express CD80 and CD86. We found that similar to BW5147-based APC, OVA_257-264_ peptide-pulsed DC2.4 cells formed a high percentage of specific cellular complex with OT-I cells, while non-specific cellular complex formations were limited (S2A Fig). After On-chip Sort-mediated sorting of each population, the OT-I/ovaAPC population again retained intact cellular complexes with a high percentage (ca. 60%), while other OT-I/nullAPC, WT/ovaAPC, and WT/nullAPC complexes were mostly disrupted, resulting in low percentages of intact cellular complexes (S2B Fig), suggesting that this phenomenon is similarly observed in professional APCs, including the DC2.4 dendritic cell line. A representative OT-I/ovaAPC micrograph is shown as S2C Fig, which was obtained without condensation post On-chip Sort-mediated sorting. Most of them were one APC with one OT-I cell (S2C Fig).

This suggests that even with gentle sorting using a microfluidics-based cell sorter, non-specific T/APC complexes did not maintain cellular complex formation. These data suggest that non-specific T/APC cellular interaction was only transient, while antigen-specific T/APC interaction was strong and stable throughout the microfluidics cell sorting process.

### Simultaneous acquisition of antigen and TCR information from an intact single T/APC complex

**Fig 5:**
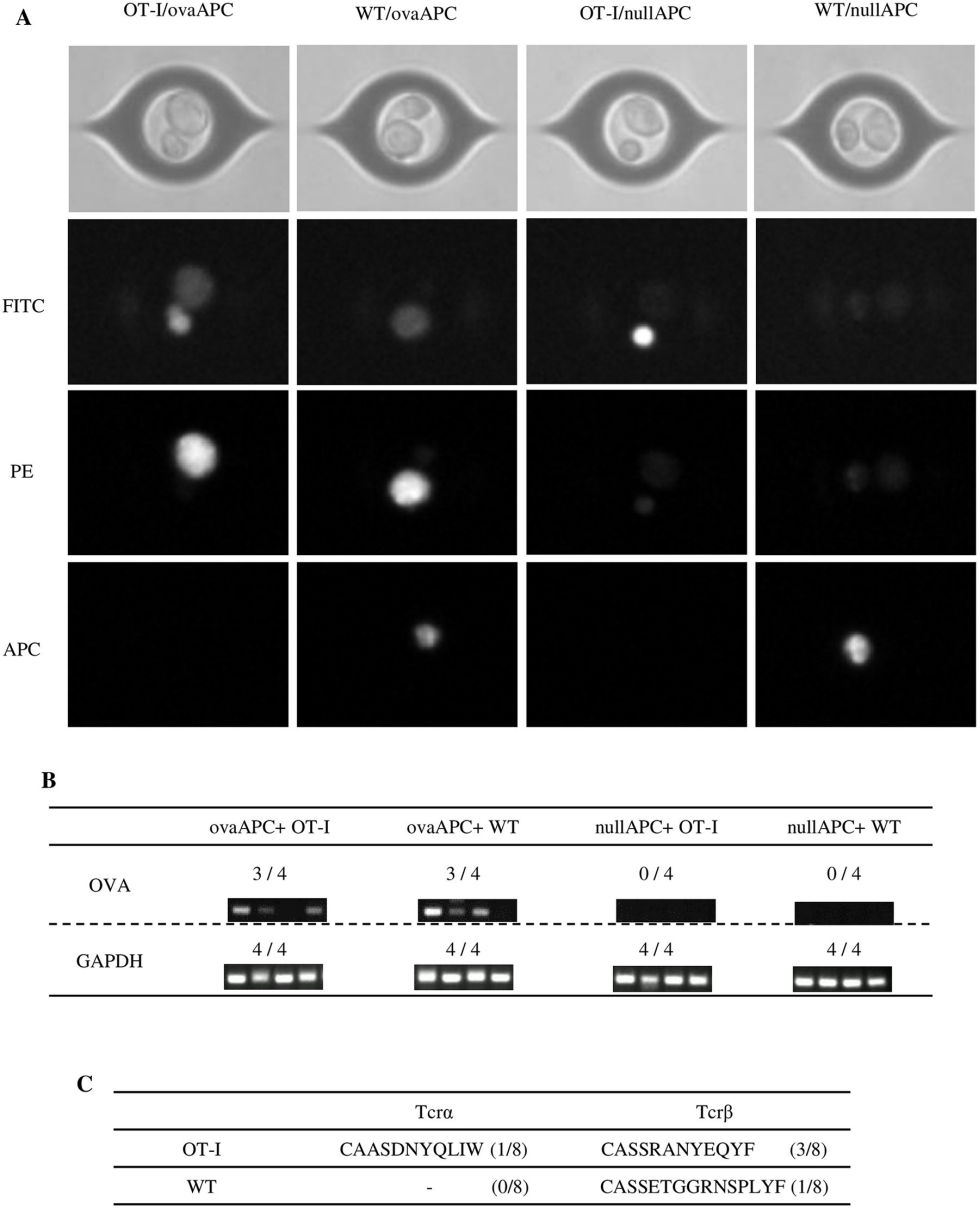
Simultaneous gene expression analysis of TCR and antigen from a single T/APC cellular complex. **(A)** Representative phase-contrast and fluorescent images of microchamber-isolated single T/APC complex using the ASONE Cell Picking System. OT-I (CFSE-labeled), WT (Far Red-labeled), ovaAPC (CMTMR-labeled), and nullAPC (CMAC-labeled) were detected using FITC channel (CFSE), PE channel (CMTMR), and APC channel (Far Red). Strong CMTMR signals leaked to the FITC channel were seen. CMAC, a blue fluorescent dye was not detected by this system. Of note, the strong PE signal of OT-I/ovaAPC and WT/ovaAPC leaked into the FITC channel. Each combination of T/APC cellular complexes was isolated using the ASONE Cell Picking System. **(B)** Four cellular complexes of each T/APC combination were isolated using the ASONE Cell Picking System. Antigen (OVA) expression was examined using RT-PCR from every single T/APC complex. GAPDH were used as a RT-PCR control. **(C)** As in (B), CDR of TCR-Vα and TCR-Vβ genes was amplified using RT-PCR, and the amplified DNA fragments were sequenced. Identified OT-I- and WT-derived CDR sequences have been shown. The numbers in the parentheses indicate successful sequence identifications per total analyzed samples.

Finally, we examined whether paired TCR and antigen sequence information were retrievable from a single T/APC cellular complex-derived mRNA. We picked up each specific/non-specific single cellular complex using a single-cell picking system (Fig 5A), and then prepared mRNA from the single T/APC complex. A representative of four samples each were then examined for OT-I/ovaAPC, OT-I/nullAPC, WT/ovaAPC, and WT/nullAPC cellular complexes. Antigen OVA genes were detected at 75% (7/8) using OVA-specific primers from ovaAPC (OVA-H2K^b^-BW5147)-containing complexes, while the OVA gene was not detected (0/8) from the nullAPC (H2K^b^-BW5147)-containing complexes (Fig 5B). TCR sequence analysis determined the OT-I TCR-Vα and TCR-Vβ CDR3 sequences as CAASDNYQLIW and CASSRANYEQYF, respectively. We retrieved the OT-I-derived TCR-Vα CDR3 sequence at 12.5% (1/8), and the TCR-Vβ CDR3 sequence from the OT-I-containing complexes at 37.5% (3/8) by using a previously reported multiplex nested PCR method [35]. We could only detect one TCR-Vβ CDR3 sequence (CASSETGGRNSPLYFT) from the WT-containing complexes. Although the efficiency of our manual mRNA analysis of a single T/APC complex was not very high, we could detect the expected antigen and TCR information at workable efficiency. This suggests that our microfluidics-based sorting of the T/APC cellular complex is useful to obtain paired information of a TCR and its cognate antigen.

## Discussion

In this study, we first quantitatively analyzed T/APC complex formation using conventional flow cytometry and demonstrated that antigen-specific T/APC interaction is more frequent and stable than antigen non-specific interaction (Figs 1 and 2). Then, we developed a microfluidics-based method to isolate the T/APC complex and demonstrated that such a microfluidics-based isolation of the intact antigen-specific T/APC complex is a useful approach to determine paired information of the antigen and TCRs (Figs 3–5), which is otherwise difficult, and thus has not been examined in the past. Simultaneous determination of this paired information is not easy because in the popularly used methods, T cell and APC have usually been analyzed separately. In general, antigen-responsive TCR determination requires prior information about the antigenic T cell epitope peptides. In contrast, antigen discovery of the T cell needs information about the pre-determined single T cell clone or the TCRα/TCRβ. In this study, we aimed for direct isolation of an intact T/APC complex and demonstrated that intact isolation of such a complex, followed by simultaneous analysis of antigen and TCR gene information, were achievable by combining microfluidics-based cell sorting and single-cell sequence technique.

T/APC complex formation has been shown to be affected by many factors, including peptide/MHC affinity or CD80/86 costimulatory molecule expression on the APCs [9, 37, 38]. High-affinity peptide/MHC and TCR interactions, namely T/APC interactions, are more stable as compared to low-affinity ones [9, 38]. Therefore, our methods can also be affected by the affinity of peptide/MHC and TCR interactions. Interestingly, low-affinity interactions rapidly detach the T cell from the APC *in vivo* and produce a release of transient effector T cells from lymph nodes, while high-affinity interactions are associated with stable T/APC interactions, which produce delayed but strong effector T cell release [9, 38], suggesting that our method is suitable for high-affinity peptide/MHC and TCR interaction detection. T/APC interaction is also influenced by CD80/86 expression on APCs [37]. Lim *et al*. have shown that the presence of CD80/86 molecules on DCs stabilizes the DC:T interaction [37]. In our case, BW5147 artificial APCs do not express CD80/86, but another APC used in this study, DC2.4 dendritic cell line has been shown to express CD80/86. In this study, both BW5147 and DC2.4 showed comparable T/APC stable complex formations, which is also dependent on the existence of cognate antigen (Fig 4 and S2 Fig). Although DC2.4 may have more stable T/APC complex formation, our results showed no obvious difference between BW5147 and DC2.4 cell lines (Fig 4 and S2 Fig). The effect of peptide affinity and costimulatory molecule expression on T/APC cellular complex formation needs to be studied further in the future.

Importantly, our results also suggested that the ability to sort an intact T/APC complex itself indicates that the isolated complex was very likely to be formed by the specific interaction between the cognate antigen peptide presented on the MHC and the antigen-specific TCR recognition (Fig 4 and S2 Fig). When the T/APC interaction was not antigen-specific, the complex formation was transient and usually detached even with the microfluidics-based sorting, which is well recognized for its low shear stress for sorting cells (Fig 4 and S2 Fig). We think this phenomenon itself is favorable for antigen and TCR discovery from bulk samples such as mouse spleen and human blood, although our study only examined the reconstituted condition by using the model system of OVA-derived SIINFEKL/H-2K^b^ antigen and OT-I-derived CD8^+^ T cells. We believe that a similar strategy is theoretically applicable for more comprehensive discovery of antigen epitope and TCR in mouse and human samples.

Although our data suggest that post microfluidics sorting, the existence of an antigen-specific T/APC interaction itself is a useful indicator of antigen-specific T/APC isolation and further analysis, at least two considerable limitations exist. First, the antigen-specific T cell frequency *in vivo* (like in mouse and human) is usually very low, ranging from 1–100 cells per 1×10^6^ cells, depending on the different antigen specificity [39, 40]. In this study, we stained each cell population with different fluorescent dyes in advance to analyze them on an individual cell basis, so that we could observe the results even with an input of 10 OT-I T cells into cell mixtures of 1×10^6^ cells each; we detected that the specific OT-I/ovaAPC complex was six to ten times higher in frequency, as compared to the OT-I/nullAPC complex (Fig 2B and 2D). However, in the physiological *in vivo* situation, antigen-non-specific irreverent polyclonal T cells exist in excess amounts as compared to antigen-specific T cells, and it is usually impossible to label the target antigen-specific T cells of interest in advance. Without the specific labeling, our approach can not differentiate between antigen-specific and -non-specific T/APC interactions by means of only the cellular complex formation itself, due to the formation of about 1% non-specific T/APC complexes (Fig 2C). Indeed, we have tried to apply our method to *in vivo* Listeria- and influenza virus-infected mouse polyclonal T cells, but currently the Listeria- and/or influenza-specific T cell and APC complex recovery has not been successful, possibly due to the above mentioned limitation in the current detection of our system. Second, TCR gene information was not efficiently retrieved using our manual single T/APC-based PCR amplification and regular Sanger sequencing method. We only detected OT-I-derived TCRα and TCRβ gene sequences in 1 and 3 samples, respectively, out of the total of 8 samples. We also tried to recover the WT TCR sequence from the WT T cell-containing single T/APC complex-derived samples, but only 1/8 TCRβ and zero TCRα sequences were obtained (Fig 5C). This may be due to our suboptimal single cell-based PCR conditions. Additionally, the low yield of TCRα sequence obtained may indicate that a dual TCRα was expressed in one T cell [41, 42]; therefore, the regular sequencing method may not work well for this purpose. These two limitations of our approach need to be addressed in future experiments, for more physiological applications.

We also recognized T/APC complex heterogeneity, especially after On-chip Sort enrichment. After sorting, the antigen-specific T cells and antigen-expressing APCs were found to be present in high density, possibly due to the interaction of one APC with two or three T cells post sorting, which can be seen in our On-chip Sort enrichment experiment (Fig 4B). This observation also has been reported in an *in vivo* experiment which used high amount of antigen-specific T cell adaptive transfer, where one cognate APC with antigen was found to be complexed with many T cells [12]. Supporting this hypothesis, our experiment in S2C Fig demonstrated that even after cell sorting, diluted T/APC complexes seemed to be mostly one APC and one T cell, suggesting that T/APC complex heterogeneity is strongly influenced by cell density-dependent cellular interactions.

Although these limitations need to be overcome in the future, our method can be applied to discover new antigens for disease and cancer-specific T cells using T/APC stable complex formation as an index. One of the potential applications of our method in the future lies in the discovery of new T cell antigens. Our BW5147-based APC can be efficiently transduced using a retroviral expression library, which easily expresses the potential antigen and corresponding HLA. Interacting cell complexes of T cells from cancer or infectious disease patients with library-expressing APCs can be picked up using our system. Thus, our system can be used for discovery of disease and cancer reactive T cells with unknown antigen specificity in samples from cancer or infectious disease patients in the future. Our method may recover the new antigen and reactive T cells as a cellular complex, and then as demonstrated in Fig 5, antigen gene information and the associated TCR sequences can be simultaneously recovered from this complex.

Summarily, in the present study, we successfully obtained a single T/APC complex without losing the specific recognition-dependent cellular interaction, which allowed us to perform a simultaneous pairwise analysis of antigen and antigen-specific TCR. Although our current method needs improvements for wider usage, we believe that some important knowledge can be acquired from this antigen-specific T/APC interaction-dependent gene information retrieval technique.

## Conclusions

The present study resulted in the successful recovery of a single antigen-specific intact T/APC complex using a microfluidics cell sorter. Moreover, the combination of a microfluidics-based flow cytometer and single-cell picking system enabled paired gene analysis of the antigen and TCR from a specific single T/APC complex. Although further work is required to confirm the validity of this approach for more physiological conditions, pair discovery of antigen and antigen-specific TCRs can be useful for future antigen-specific T cell therapy or TCR gene therapy for autoimmune diseases, cancer, and infection.

## Supporting information

S1 Fig Non-specific T/APC complex sorting using FACSAria^™^ II. The CMTMR/Far Red complex, which is one of the non-specific T/APC complexes, maintained its complex formation in only 0.365% of the total population, post sorting with the FACSAria^™^ II.
(PDF)

S2 Fig OVA_257-264_ peptide-pulsed DC2.4 cells also formed a stable cellular complex with OT-I cells. The experimental protocol was the same as that in Fig 4, except in this case DC2.4 cells were used as the APCs instead of BW5147 cells. DC2.4 cells were pulsed with 5 μg/mL of OVA_257-264_ peptide (257DC2.4) or not (nullDC2.4), and then processed similar to BW5147 cells. **(A)** On-chip Sort plot before sorting. **(B)** Re-analysis plot post On-Chip Sort-mediated sorting using Novocyte^®^ flow cytometer. **(C)** A representative micrograph of a OT-I/254DC2.4 cellular complex sorted using On-chip Sort. These cells were not centrifuged post On-chip Sort and directly observed using a fluorescence microscope.
(PDF)

S1 Raw images (PDF)
