# Supplementary material for: Microfluidics sorting enables the isolation of an intact cellular pair complex of CD8+ T cells and antigen-presenting cells in a cognate antigen recognition-dependent manner
Source: PLoS One. 2021 Jun 14;16(6):e0252666. doi: 10.1371/journal.pone.0252666 (PMC8202920; doi:10.1371/journal.pone.0252666)
Supplement: S1 Fig — The CMTMR/Far Red complex, which is one of the non-specific T/APC complexes, maintained its complex formation in only 0.365% of the total population, post sorting with the FACSAria™ II. (PDF) [file pone.0252666.s001.pdf]

Supplementary Fig. 1

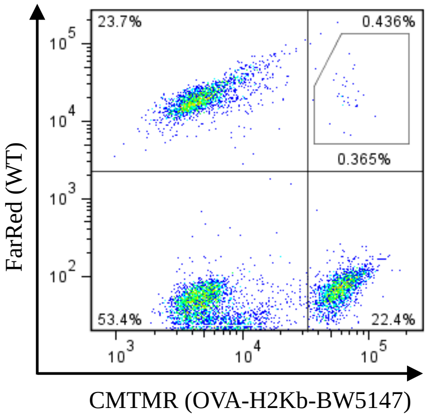

Supplementary Figure 1. Non-specific T/APC complex sorting by FACSAriaII
The CMTMR/FarRed complex which is one of non-specific T/APC kept complex formation at only 0.365% of total populations after soring by FACSAriaII.
