# Supplementary material for: Microfluidics sorting enables the isolation of an intact cellular pair complex of CD8+ T cells and antigen-presenting cells in a cognate antigen recognition-dependent manner
Source: PLoS One. 2021 Jun 14;16(6):e0252666. doi: 10.1371/journal.pone.0252666 (PMC8202920; doi:10.1371/journal.pone.0252666)
Supplement: S2 Fig — The experimental protocol was the same as that in Fig 4, except in this case DC2.4 cells were used as the APCs instead of BW5147 cells. DC2.4 cells were pulsed with 5 μg/mL of OVA257-264 peptide (257DC2.4) or not (nullDC2.4), and then processed similar to BW5147 cells. (A) On-chip Sort plot before sorting. (B) Re-analysis plot post On-Chip Sort-mediated sorting using Novocyte® flow cytometer. (C) A representative micrograph of a OT-I/254DC2.4 cellular complex sorted using On-chip Sort. These cells were not centrifuged post On-chip Sort and directly observed using a fluorescence microscope. (PDF) [file pone.0252666.s002.pdf]

Supplementary Fig. 2

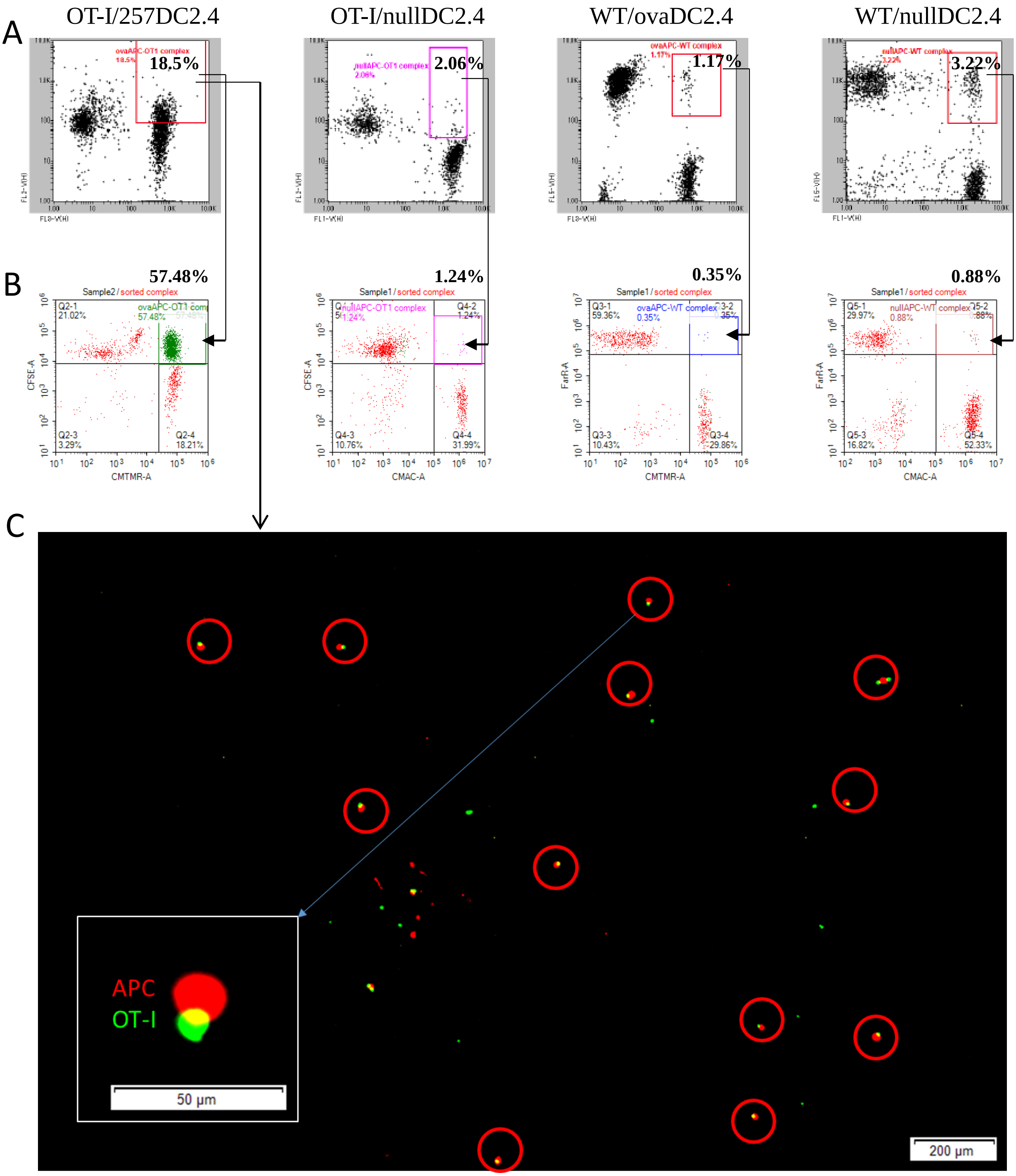

Supplementary Figure 2. OVA257-264 peptide pulsed DC2.4 also showed stable cellular complex with OT-I cells

Experiment was performed as in Figure 4 by using DC2.4 instead of BW5147 cells as APC. DC2.4 cells were pulsed with 5μg/mL of OVA257-264 peptide (257DC2.4) or not (nullDC2.4), and then used similarly to BW5147 cells. (A) On-Chip sort plot before sorting. (B) Re-analysis plot after On-Chip sort sorting by Novocyte flowcytometer. (C) A representative micrograph of OT-I/254DC2.4 cellular complex sorted by On-chip sort. These cells are not centrifuged after On-chip sort and directly observed by fluorescent microscope.
