# Supplementary figures and images for: Microfluidics sorting enables the isolation of an intact cellular pair complex of CD8+ T cells and antigen-presenting cells in a cognate antigen recognition-dependent manner
Source: PLoS One. 2021 Jun 14;16(6):e0252666. doi: 10.1371/journal.pone.0252666 (PMC8202920; doi:10.1371/journal.pone.0252666)

Original Gel Image for Fig 5B

OVA product : 157 bp  
GAPDH product : 172 bp

Low Molecular Weight  
DNA Ladder (NEB)

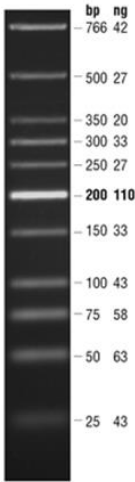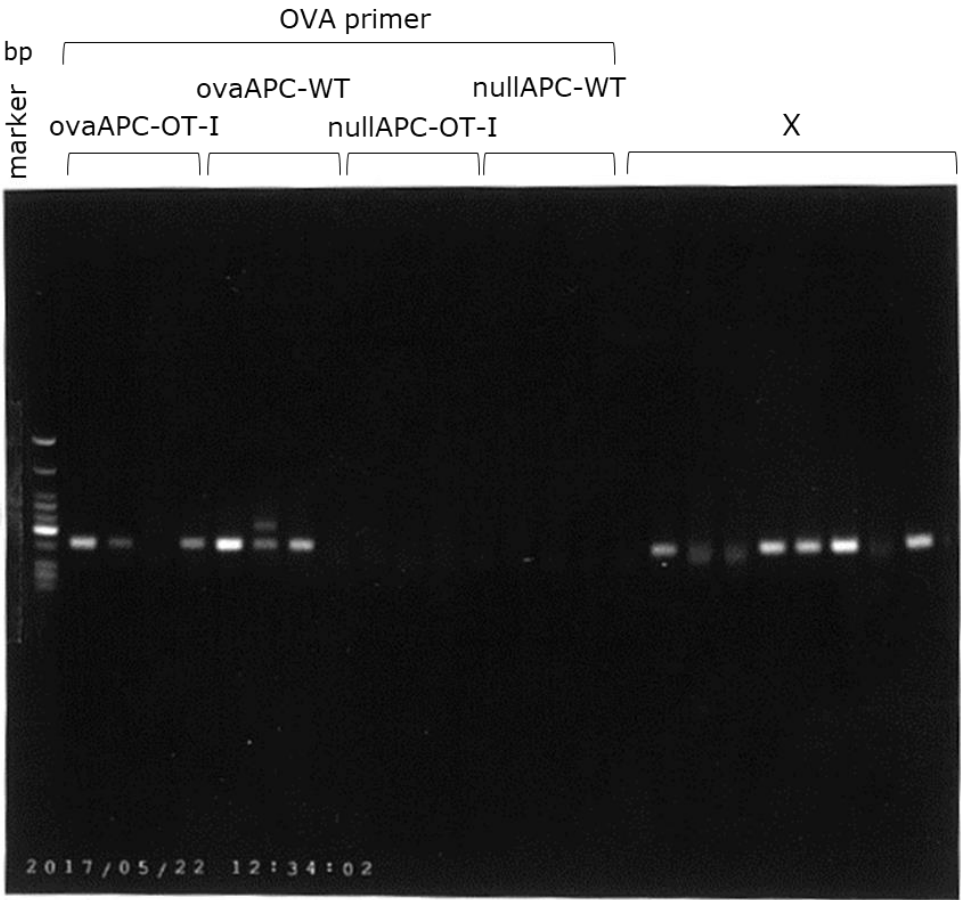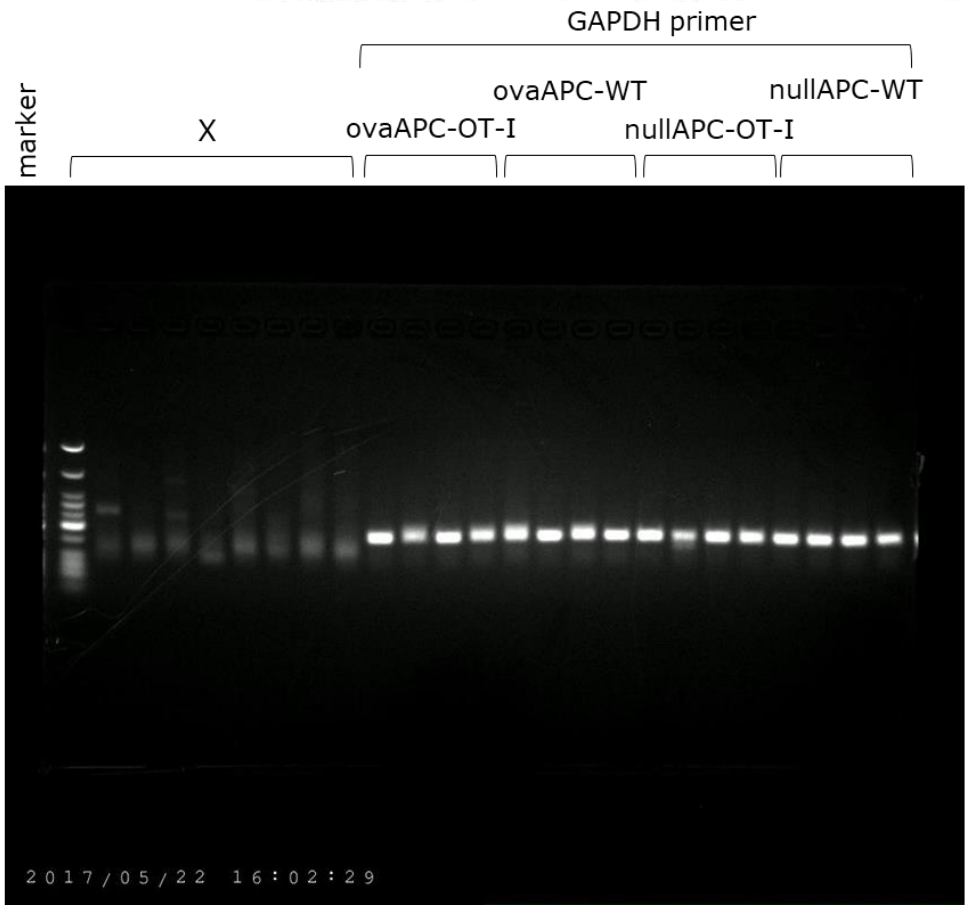

Supplement: S1 Raw images — (PDF) [file pone.0252666.s003.pdf]
